# Wild epiphytic Bangladeshi orchids *Cymbidium aloifolium* (L.) Sw. and *Papilionanthe teres* (Roxb.) Lindl. potentially modulates the immune functions in Swiss albino mice

**DOI:** 10.5455/javar.2021.h537

**Published:** 2021-09-20

**Authors:** A. M. Abu Ahmed, Md. Atiar Rahman

**Affiliations:** 1 Department of Biochemistry and Molecular Biology, University of Chittagong, Chittagong, Bangladesh; 2 Department of Genetic Engineering and Biotechnology, University of Chittagong, Chittagong, Bangladesh

**Keywords:** Cymbidium aloifolium, Papileoanthe teres, immunomodulatory activity, SRBC, Swiss albino mice

## Abstract

**Objective:**

This research investigated the immunomodulatory potentials of two medicinally important wild epiphytic Bangladeshi orchids *Cymbidium aloifolium* and *Papilionanthe teres* using Swiss albino mice.

**Materials and Methods:**

Orchid extracts were prepared using a cold methanol extraction procedure. To assess the immunomodulatory action, Swiss albino mice of either sex weighing 25–35 gm were divided into five groups each with six animals. Sheep red blood cells (SRBC) of 0.5 × 10^9^ cells/ml were used to immunize all mice on the 7th day, and a booster dose of the same quantity of SRBC was given on the 11th day of the experiment. After 14 days of oral treatment with 100 and 200 mg/kg bw of orchid extract, the mice were sacrificed to collect serum and organs. Hematological assays, delayed type of hypersensitivity assays, phagocytic index (PI), and histopathological investigations were used to assess *in vivo* immunomodulatory efficacy.

**Results:**

The body weight changes of the experimental animals were considerably greater at 100 mg/kg bw than at a higher dose (200 mg/kg bw). There was a substantial improvement of relative organ weights of the thymus and spleen at the low dose, but no effect on kidney weights was evident. The liver weight increased significantly (*p* < 0.05) at both doses. Total neutrophil, leukocyte, and lymphocyte counts, hemoglobin percentage, delayed hypersensitivity reaction, and PI were all significantly (*p* < 0.05) increased in mice receiving the lower dose. In contrast to the control group, the higher dose reduced immunological response, suggesting the negative influence of a higher dose of extracts on the immune reaction.

**Conclusions:**

The results demonstrate that orchid extracts can potentially modulate the innate immune system in the experimental animal.

## Introduction

It is vital to maintain the balance of immune systems as it is a complex protective system that defends vertebrates from infections, trauma, environmental contaminants, and illnesses that alter homeostasis. In order to guard against undesired and alien invaders, the innate and adaptive immune systems generate and mediate immune responses [1,2]. Medicinal plants play an essential role in preventing various infections through modulating immune systems. Some plants have anti-cancer, anti-inflammatory, and anti-stress effects by modulating immune functions [3]. Recently, different medicinal plants were tested for their potential antiviral activity, especially severe acute respiratory syndrome coronavirus-2 (SARS-CoV-2). *Azadirachta indica*, *Allium sativum*, *Andrographis paniculata*, *Calotropis gigantea*, *Curcuma longa*, *Glycyrrhiza glabra*, *Ocimum sanctum*, *Withania somnifera*, *Zingiber officinale*, *Tinospora cordifolia*, and *Moringa oleifera* are a few examples of plants with antiviral and immunomodulatory properties [4]. Additionally, their unique phytochemicals such as flavonoids, alkaloids, quercetin, lignans, kaempferol, luteolin, saponins, apigenin, catechins, and polysaccharides inhibit viral entry, destroy nucleocapsid and genetic material, and inhibit virus replication. As a result, these may be used to combat the devastating SARS-CoV-2 virus, which is presently spread across the globe and poses a significant threat to humanity [5,6]. Consequently, their application is growing dramatically. Natural medicines influence the immune system by inhibiting or activating innate and adaptive immune cells [7]. Immune modulation is necessary for the body’s natural immunity to function. Immunomodulatory substances are currently being sought to treat various infections by enhancing natural resistance [8,9].

Moreover, as a possible therapeutic measure, natural products have become an acceptable approach nowadays. Immunological defense systems may involve complex interactions between nonspecific and specific immunological responses, immune suppression and stimulation of immunocompetent cells, and the influence of endocrine and alternative processes [10]. T and B lymphocytes are the principal or supplemental immunostimulatory cells; enhanced phagocytosis by macrophages and granulocytes is critical for immunostimulation. Activation of macrophages may allow stimulating agents to remain in contact with reactive cells. T-lymphocytes stimulation is the second most potent role, which may be achieved either directly or indirectly via macrophages [11].

Orchids, often known as magnificent miniatures, are Orchidaceae family flowers with amazing beauty and numerous patterns. Orchids may be found worldwide, although they are more common in humid tropical and subtropical climates [12]. It has a wide range of ethnobotanical uses and is commonly used for decorative purposes. Although Bangladesh has a large number of orchid species, the majority of them are wild epiphytic and terrestrial orchids with therapeutic potentials. However, their medicinal benefits, chemical composition, and pharmacological uses are very little known [12–14]. They contain phenolics, glycosides, flavonoids, polysaccharides, alkaloids, tannins, and terpenoids, all of which have different metabolic activities that are potentially helpful to human health, including anti-inflammatory, antioxidant qualities that strengthen the immune system [15].

*Cymbidium aloifolium* (CAME) (L.) Sw. and *Papilionanthe teres* (PTME) (Roxb.) are epiphytic wild orchids that grow on tree trunks and have great medicinal value. They are found across Bangladesh, as well as in South Asia and Southeast Asia. CAME contains various bioactive compounds, including phenanthrenes: coelonin, 6-methoxycoelonin, aloifol I and II, and PTME also have various types of compounds such as eucomic acid, malic acid, and vandateroside II, all of which have potential biological activity [16]. Folklore claims that CAME is purgative and emetic in nature. The root powder is used to cure paralysis. The tribal people of the Chittagong hill tracts region use leaf extract to treat boils and fever. Aerial roots that have been pasted together are utilized to join fractured bones. Purgatives, emetic, tonics, earache, burns, and sores are all treated with the whole plant. The plant is mashed with ginger and the resulting water extract is used to treat chronic diseases, eye weakness, vertigo, and paralysis. It contains two biologically active phytochemicals, dihydrophenanthrene and bibenzyls, replaced with phenanthraquinone [13]. PTME stems and leaves are used to increase blood flow and decrease edema. Fractured bones are treated with a paste made from the plant [16]. However, evidence-based scientific researchers have hardly been undertaken to explore their effects in immunomodulation. The purpose of this study was to determine the immunomodulatory effects of methanolic extracts of CAME and PTME using a Swiss albino mouse model.

## Materials and Methods

### Chemicals and reagents

The necessary chemicals such as methanol, dextrose, citric acid monohydrate, calcium chloride, potassium chloride, trisodium citrate dehydrate, disodium hydrogen phosphate, sodium chloride, magnesium chloride, potassium dihydrogen phosphate, hematoxylin, xylene, and eosin were procured from Sigma-Aldrich (St Luis, MO). All of the chemicals used in the experiment were of analytical grade.

### Sample collection and identification

The orchid samples were collected from Ukhiya on Teknaf-Cox’s Bazar highway in Cox’s Bazar district, and taxonomical identification was confirmed by taxonomist Professor Dr. Sheikh Baktear Uddin, Department of Botany, University of Chittagong. For future reference, the sample specimens (Accession No AMNPR-HE103 and 104) are preserved in the herbarium of the Laboratory of Alternative Medicine and Natural Product Research, Department of Biochemistry and Molecular Biology.

### Extract preparation

The collected fresh samples were cleaned with tap water and dried under the shade at room temperature. Then, pulverization of dried samples was carried out using a mechanical grinder and stored in airtight polythene bags for further use. The resulting powder of these samples was converted to crude extract with methanol at room temperature for up to 48 h. Colored liquid extracts were formed, then evaporated methanol and concentrated to dryness under reduced pressure using a rotary evaporator (RE200, BIBBY Sterling Ltd., England) to form sticky semi-solid crude extracts. The extracts were preserved at 4°C in the refrigerator and used for further screening. Before that, using a digital balance, the dried pure extracts were weighed separately and the yield was measured with the following formula: 

% yield = (weight of dried extract/total amount of powder) × 100.

### Experimental design

For this study, Swiss albino mice weighing 25–35 gm of both sexes were collected from Jhangirnagar University, Savar, Dhaka. The collected animals were acclimatized with the usual food and water in laboratory conditions for a week then separated into five groups with six animals each [10]. For a week, the mice were fed and watered normally. Male and female mice were housed separately before and throughout the treatment to prevent pregnancy. OECD (The Organization for Economic Co-operation and Development) guidelines for animal studies and animal welfare were adapted to carry out the *in vivo* experiments with Swiss albino mice at the Department of Biochemistry and Molecular Biology, University of Chittagong. The University of Chittagong’s Faculty of Biological Sciences ethics committee approved this work (ERB-CU/2018-03). Experimental animals were categorized into the following groups:

Group A received normal water and 5% DMSO (Dimethyl sulfoxide) for 14 days as a control group.

Group B_1_ received 200 mg/kg bw of CAME for 14 days as a treatment group.

Group B_2_ received 100 mg/kg bw of CAME for 14 days as a treatment group.

Group C_1_ received 200 mg/kg bw of PTME for 14 days as a treatment group

Group C_2_ received 100 mg/kg bw of PTME for 14 days as a treatment group.

### Antigen preparation/immunization

The young, mature, healthy sheep were selected for collecting blood provided by Chittagong Veterinary and Animal Science University. The blood was mixed with sterile Alsever solution that was prepared with 2 gm of dextrose, 0.8 gm of trisodium citrate dehydrate, 0.055 gm of citric acid monohydrate, and 2.1 gm (1:1) of sodium chloride. The red blood cell was then centrifuged for 5 min at 1,600 *g* to settle at the bottom of the test tube. After discarding the supernatant, sheep red blood cells (SRBC) were washed in PBS (Phosphate-buffered saline; pH 7.2). SRBC was adjusted to 0.5 × 10^9^ cells/ml. The first immunization dose was given to all animals on the 7th day using a 1 ml syringe containing 0.5 × 10^9^ cells/ml of SRBC. The booster dose was given on the 11th day with the same amount of SRBC as a booster dose.

### Body and lymphoid organ weight

The mice were sacrificed 24 h after the last dose of the extract (14th day). Before that, all animals’ body weights were measured. Blood was also taken from the heart punctured using a normal syringe. Haemocytic and differential counts, and hemoglobin measured were carried out. The liver, thymus, spleen, and kidneys were carefully separated, and their relative weight was calculated. The organs were fixed with phosphate-buffered formalin after being rinsed with 0.9% saline for histological studies later.

### Hematological parameters

Blood was collected from the heart with a needle and syringe and kept in heparinized vials coated with ethylenediaminetetraacetic acid. The collected blood was subjected to several hematological assays, including total count, WBC, RBC, neutrophil, lymphocyte, monocyte, eosinophil, basophil, and hemoglobin percentage.

### Differential count of white blood cells

To count different WBC, a blood smear was made onto a clean, sterile glass slide. It was then dried and fixed for 5 min in methanol. After drying in the oven, the slide was submerged in Field’s stain solution B for 5 sec. After that, it was cleaned with double distilled water and dried. After that, it was stained for 20 sec with Field’s stain solution A. The slide was cleaned with double distilled water after staining and dried again. A fluorescent microscope (Euromex IS.3153-PLi/6) was then used to examine a stained slide.

### Delayed type hypersensitivity (DTH) response

Mice were sensitized by injecting 0.15 ml of 1.25 × 10^9^ SRBC/ml subcutaneously into the right hind footpad. A similar volume of saline was given to the healthy control group at the same time. After 24 h of sensitization, the swelling edema in the footpad thickness was measured using a spheromicrometer (0.01 mm pitch). The difference in footpad thickness calculated the DTH response before and after the adjustments.

### Phagocytic activity of macrophages

The experiment was carried out with minor modifications according to Ramesh and Padmavathi’s [17] technique. Capillary blood was harvested from a healthy young man, and 0.2 ml was flooded on a sterile clean glass slide (Merck, Germany) and stretched to 1.5 cm for the experiment. After allowing the blood to coagulate at 37°C for 25 min, the lump was carefully separated with sterile common saline. It is important not to disrupt the neutrophils attached to the surface. Although most of the blood components were washed free, the polymorphonuclear leukocytes (PMNs) were found to bind to the slide wall. After flooding two doses of the methanolic extracts (0.1 ml) above the PMN layer on the slides, they were placed in an incubator at 37°C for 15 min, and then 100 ml of *C. albicans* cell suspension with a concentration of 10 × 10^6^ cells/ml was added. The slides were then incubated for another 60 min at 37°C. The slides were washed twice with sterile normal saline after incubation. Methanol was used to fix the slides for 5 min. The slides were soaked with a diluted Giemsa stain and left undisturbed for 25 min. Hank’s balanced salt solution was used to remove the surplus stain, which was then air-dried. The experiment was repeated thrice. The slides were then examined using an oil immersion (×100) objective. Using physical criteria, the average number of *Candida* cells phagocytosed by PMNs on the slide was determined microscopically for 100 granulocytes. The following method was used to calculate phagocytic activity as a phagocytic index (PI): 

PI = AB

where “A” represents the proportion of *Candida* cells ingesting phagocytes and “B” represents the quantity of *Candida* cells ingested/phagocytes [18].

### Histopathological analysis

All sacrificed mice spleens were removed and rinsed with 0.9% normal saline before being preserved in 10% buffered formalin. To dehydrate the tissues, a series of increasing ethanol concentration solutions (70% for 5 min, 80% for 5 min, 90% for 5 min, 95% for 5 min, 100% for 3 min) was used before being cleansed in xylene. The specimen was infiltrated using molten paraffin and allowed to solidify to a consistency that cuts into a microtome of 5 μm in thick pieces. Hematoxylin and eosin (H&E) were used to stain the tissue. After that, the tissue section was dehydrated again with ascending graded ethanol of 70%, 80%, 90%, and 100% for 1 min each and finally passed through xylene solutions thrice. Lastly, the histological sections were observed under a fluorescence microscope (Euromex IS.3153-PLi/6).

### Statistical analysis

All data were analyzed through one-way analysis of variance (ANOVA) with Dunnett’s test and two-way ANOVA, followed by Tukey’s multiple comparison tests using GraphPad Prism Version 8.0.2. Values are expressed as mean ± standard deviation (SD). *p*-values (*p* < 0.05) were considered as statistically significant. 

## Results

### Effect of plant extracts on body and organ weight

The body weight of the normal control group and both doses (200 and 100 mg/kg bw) of the treatment groups were gained during the experimental period. However, the animal at the highest dose (200 mg/kg bw) showed lower body weight than the lowest (100 mg/kg bw). The body weight was a significant difference between the lowest and highest doses of CAME and PTME (*p* < 0.05). No significant thymus relative weight was recorded for PTME in both doses except for CAME (200 mg/kg bw). However, a substantial increase (*p* < 0.05) in relative spleen weight was recorded at the lowest dose (100 mg/kg bw) for both samples. Between the two doses, there was no significant variation in relative kidney weight. In terms of the liver, substantial impacts were observed at the higher dose (200 mg/kg bw for) of both samples, although moderate effects were detected at the lower dose (100 mg/kg bw) when compared to normal control (Table 1).

### Delayed hypersensitivity test

**Figure 1.**
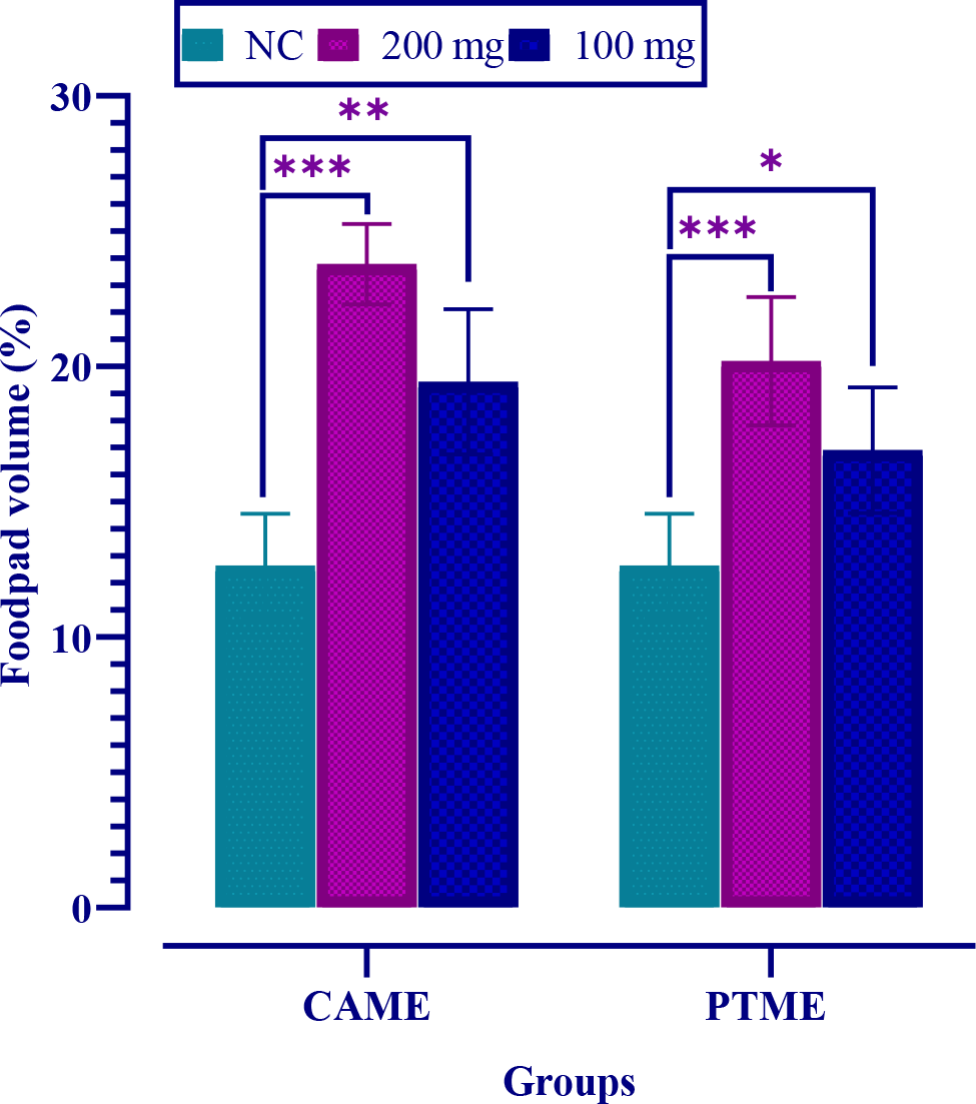
Albino mice were fed with two concentrations (200 and 100 mg/kg bw) of CAME and PTME to develop delayed-type hypersensitivity. Values are expressed as mean ± SD, where *n* = 6 animals are compared with two-way ANOVA, followed by Tukey’s multiple comparison test. *p < 0.05, **p < 0.01, and ***p < 0.001, and ns = no significance compared to the normal control (NC).

Delayed hypersensitivity was observed for every group of animals induced by SRBC. The remarkable increase in paw volumes was recorded for both doses administrated in rat groups. The maximum paw-volume increase was recorded as 23.78 ± 1.24% for CAME and 20.19 ± 1.24% for PTME at 200 mg/kg bw groups. On the other hand, a moderate increase in paw volume (19.43 ± 1.25% for CAME and 16.90 ± 1.24% for PTME) was noted for lower doses (100 mg/kg bw). Values were statistically significant (*p* < 0.05) compared to the normal control group (Fig. 1).

**Table 1.** Effect of plant extracts on the body and relative organ weight of mice.

Groups	Initial body Wt.	Final body Wt.	% of body wt. changes	Thymus	Body organ weight ratio	Liver	Spleen
					Kidney		
A	29.4 ± 1.34	30.8 ± 1.48	4.77 ± 1.87	0.11 ± 0.02	1.16 ± 0.16	5.32 ± 1.21	0.61 ± 0.05
B_1_	25.00 ± 0.71	25.60 ± 1.08	2.37 ± 1.64*	0.12 ± 0.02**	1.31 ± 0.25	6.18 ± 0.60	0.67 ± 0.07
B_2_	23.20 ± 1.92	24.48 ± 2.06	5.52 ± 1.03*	0.12 ± 0.02	1.49 ± 0.08	5.67 ± 0.40**	0.81 ± 0.18*
C_1_	29.00 ± 2.24	30.00 ± 2.24	3.45 ± 0.28*	0.11 ± 0.02	1.29 ± 0.09	6.24 ± 0.34	0.59 ± 0.07
C_2_	26.40 ± 1.52	27.52 ± 1.71	4.24 ± 1.67*	0.14 ± 0.03	1.30 ± 0.15	5.77 ± 0.51*	0.74 ± 0.07*

Values are expressed as mean ± SD, where *n* = 6 animals were compared with one-way ANOVA, followed by Tukey’s multiple comparison test.

* *p* < 0.05,

** *p* < 0.01.

### Hematological studies

**Figure 2.**
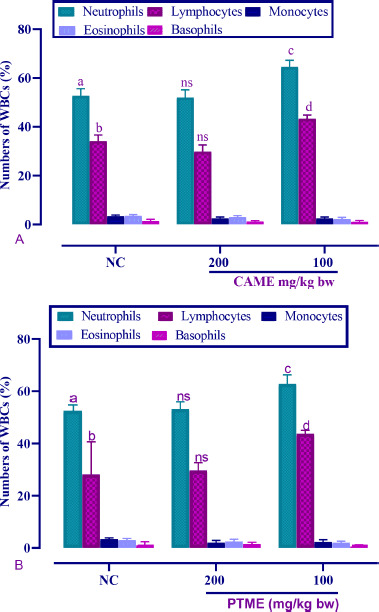
Graph showing the differential count of albino mice’s blood fed with (A) CAME and (B) PTME. Values are expressed as mean ± SD, where *n* = 6 animals are compared with two-way ANOVA, followed by Tukey’s multiple comparison test. ^a, b, c, d^significant difference between each other (*p* < 0.05) and ns indicates no significance compared to the normal control (NC).

When CAME was compared to the control group, lymphocyte and monocyte elevations were detected at the lowest dose (100 mg/kg bw), but the maximum dose (200 mg/kg bw) did not demonstrate a meaningful increase. The neutrophil decreased at a higher dose but increased at the lower one (Fig. 2A). When compared to the control group, the lowest dose (at 100 mg/gm bw) of PTME significantly enhanced the number of lymphocytes and neutrophils. However, the percentage of monocytes, basophils, and eosinophils was decreased compared to the higher dose (200 mg/kg bw) in both samples (Fig. 2A and B).

**Figure 3.**
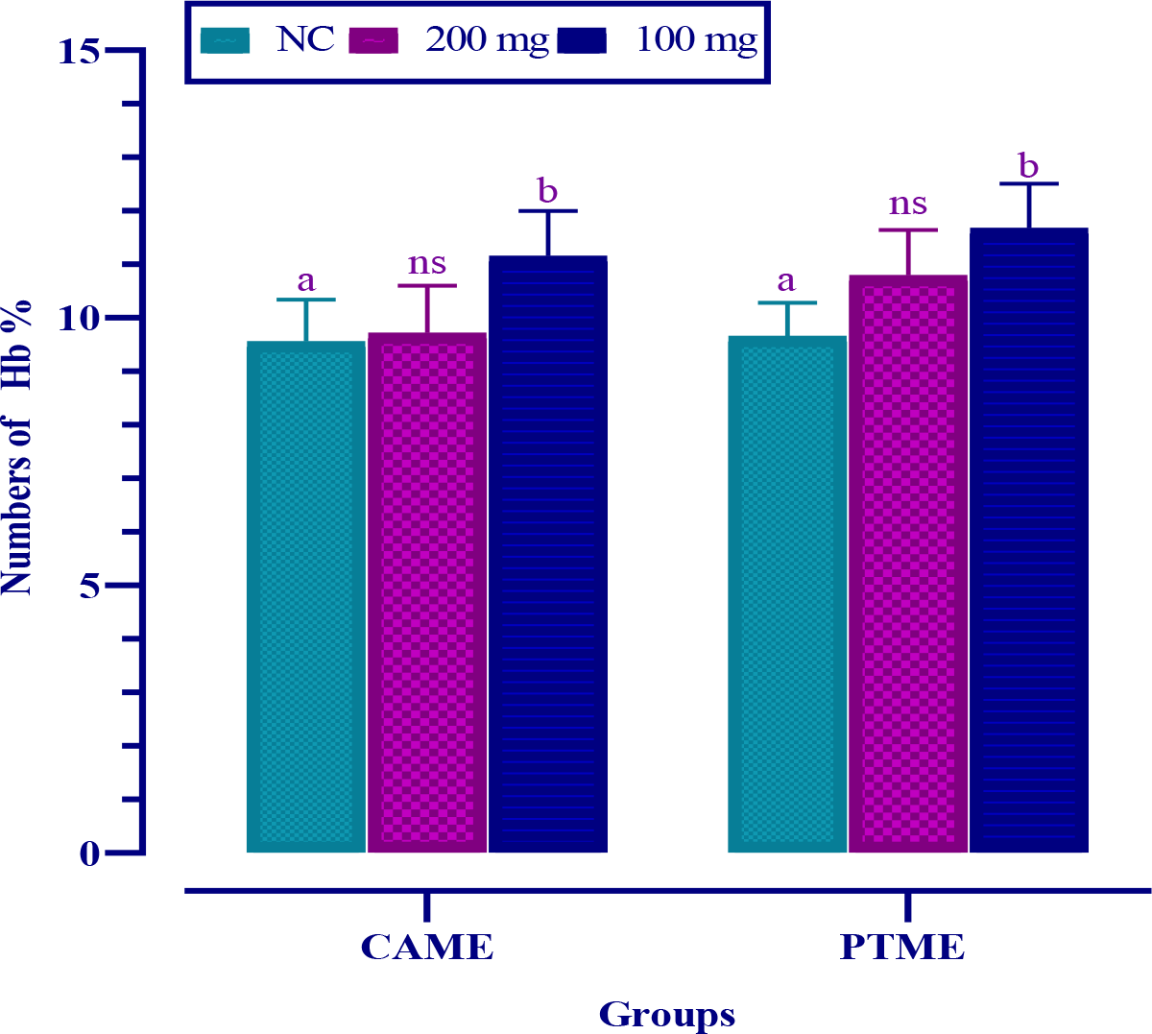
Percentage of hemoglobin of albino mice’s blood fed with CAME and PTME*.* Values are expressed as mean ± SD, where *n* = 6 animals are compared with two-way ANOVA, followed by Tukey’s multiple comparison test. ^a^significant difference at *p* < 0.05 level and “ns” is not significant compared to the normal control (NC).

**Figure 4.**
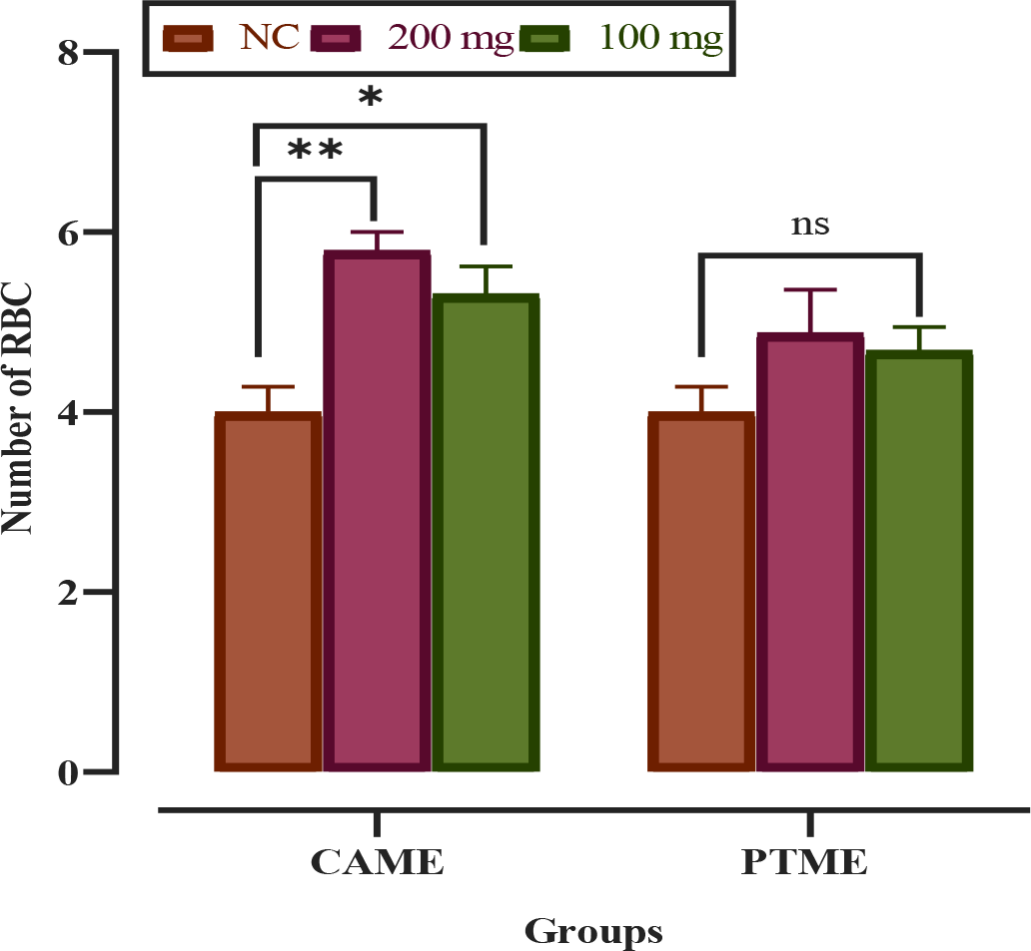
Graph showing the comparative number (%) of red blood cells of albino mice fed with CAME and PTME. Values are expressed as mean ± SD, where *n* = 6 animals are compared with two-way ANOVA, followed by Tukey’s multiple comparison test. ^a^significant difference at *p* < 0.05 level and “ns” is not significant compared to normal control (NC).

**Figure 5.**
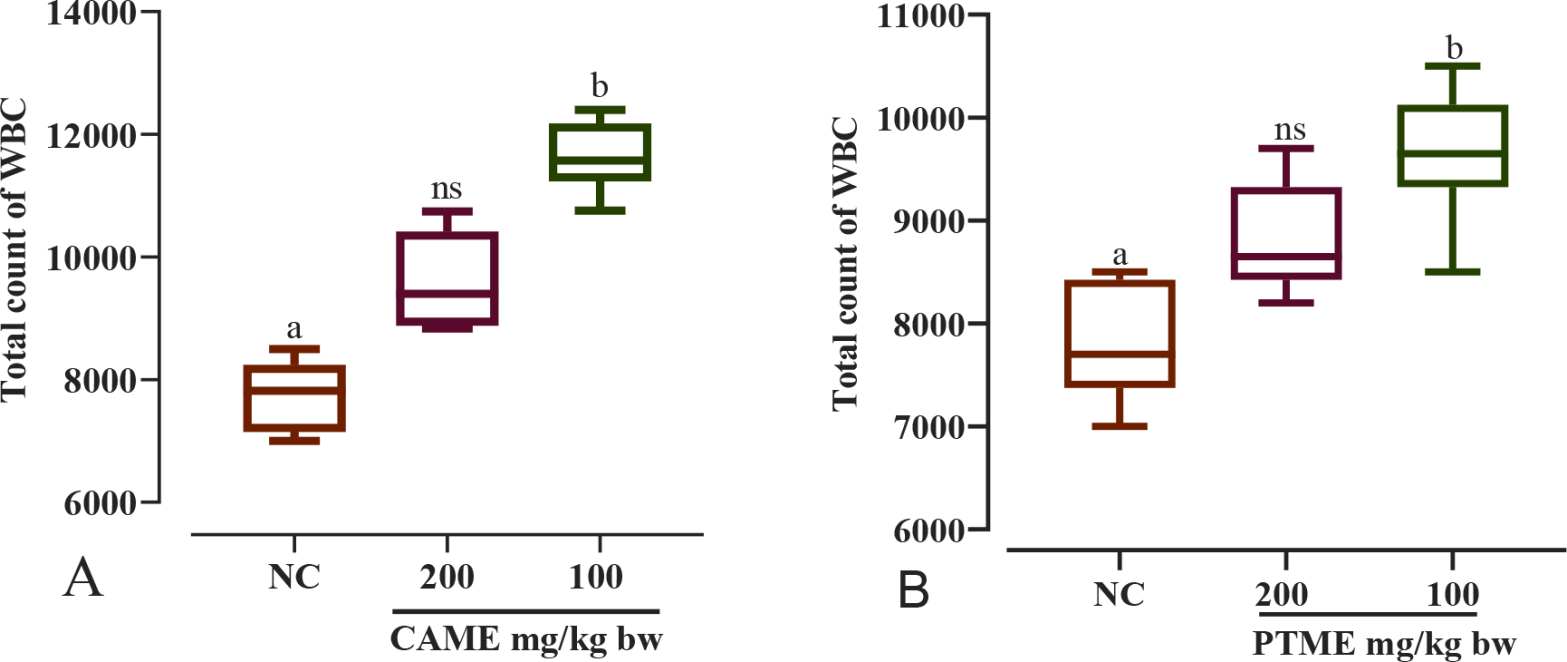
Graph showing the total number of WBC of albino mice fed with (A) CAME and (B) PTME. Values are expressed as mean ± SD, where *n* = 6 animals are compared with one-way ANOVA, followed by Tukey’s multiple comparison test. a = *p* < 0.05 and ns = no significance compared to the normal control (NC).

Compared to the control group, a significant (*p* < 0.05) increase in hemoglobin was noted as 11.1 mg/dl for CAME and 12.10 mg/dl for PTME at the lower dose of 100 mg/kg bw. On the contrary, the higher dose (200 mg/kg bw) could not increase the hemoglobin level in most cases (Fig. 3). CAME significantly increased the number of RBCs by all doses compared to the control group, but PTME did so only with the lower dose (Fig. 4). Reversely, the total WBC count was remarkably elevated by CAME and PTME at the lower dose only compared to the normal control group (Fig. 5A and B).

### Effect of plant extracts on phagocytic activity of macrophages

**Figure 6.**
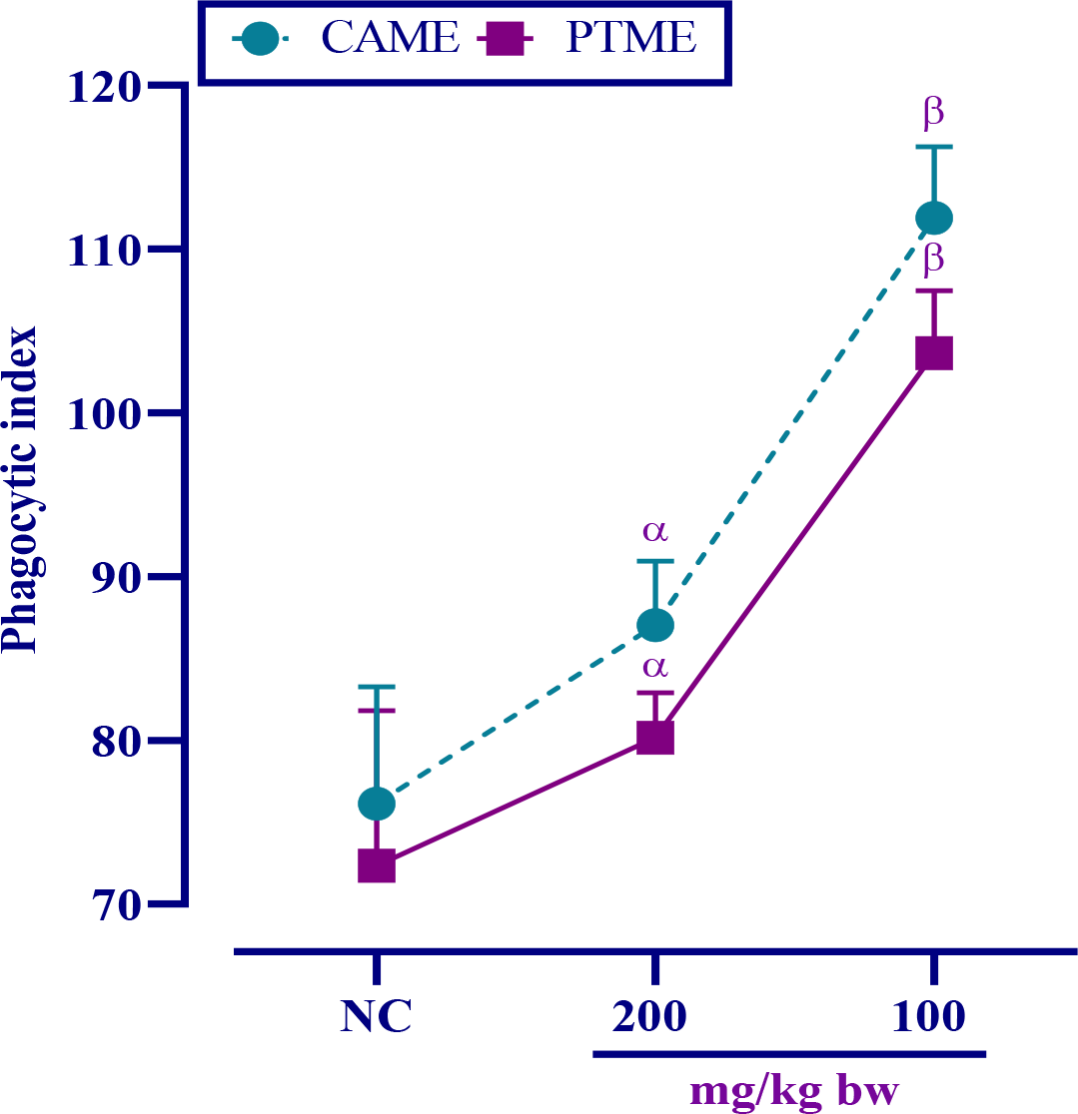
Effect of CAME and PTME on the nonspecific immunity as assessed by the PI. Data are means ± SD, where *n* = 6 animals; *α* = *p* < 0.05 and *β* = *p* < 0.001 compared with the control animals (significantly different).

The effects of CAME and PTME are shown in Figure 6. At a lower dose, CAME and PTME had, respectively, shown PI of 115.32 ± 2.48 and 102.43 ± 7.03, which were correspondingly 88.69 ± 3.11 and 81.32 ± 4.95 for the higher dose. However, in both cases, the values were significant (*p* < 0.05) compared to normal control, and a lower dose was found to be better than the higher dose to maintain the PI. 

### Histopathological studies of the spleen

**Figure 7.**
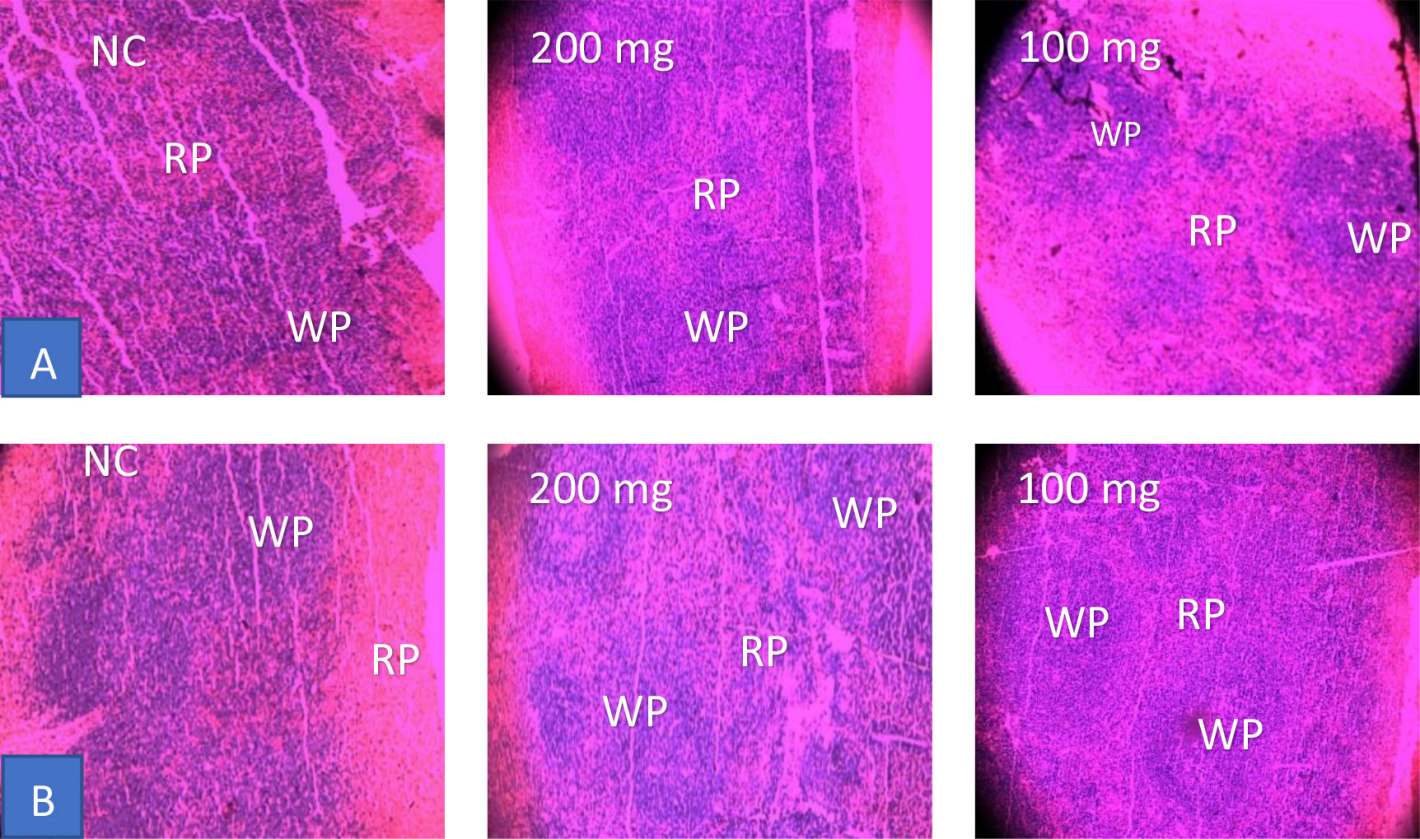
Histopathological images of the spleen taken from intact SRBC control and treated mice (WP = white pulp, RP = red pulp, and NC = normal control). (A) CAME-induced mice distorted (NC), moderate (200 mg) and well-organized (100 mg) WP and RP and (B) PTME-induced mice distorted (NC), moderate (200 mg) and well-organized (100 mg) WP and RP are seen.

According to microscopic sections of the spleen stained with H & E, the slightly distorted lymphoid architecture minimized lymphoid follicles, evident with large white pulp showed by CAME in an SRBC induced control. Moreover, a more precise and organized white pulp over the red pulp was found at the lowest dose (100 mg/kg bw). Additional damage was seen at the higher dose (200 mg/kg bw) with a slight distortion of lymphoid architecture (Fig. 7A). Similarly, both doses of PTME were found to restore the well-defined white pulp instead of large and defused red pulp in SRBC-induced control (Fig. 7B).

## Discussion

The body’s immune system is vital for fighting against various infectious microorganisms and tumors, cancer, and many more diseases. The immune system’s balance plays a pivotal role in maintaining sound health; if this equilibrium is disrupted, the immune system will not defend itself against toxic substances [19]. The search for immunomodulatory drugs with fewer side effects that may be administered for longer periods of time to produce sustained immunological activation for disease prevention has been forced by the adverse effects and expensive demand of allopathic drugs. The medicinal plant can modulate the immune system to achieve different forms of traditional chemotherapy for various illnesses, particularly when the host resistance system needs to trigger in the presence of a cooperated immune response needed for autoimmune disorders [20]. Immunostimulating agents are used to treat a range of disorders in which the immune system has been suppressed by drugs or environmental factors. To counteract the immunosuppressive symptoms of stress, chronic illnesses, and disorders of compromised immune responsiveness, medications that can improve the immune system are in high demand [21].

Two epiphytic wild medicinal orchids were chosen for this investigation to assess their immunomodulatory functions. The mice’s body weight and relative vital organs’ weight, such as liver, are affected when CAME and PTME are administered. However, natural chemicals restore organ mass and activity, as evidenced by the identical relative mass of treated and control livers at a lower extract dose, implying natural liver activity. There were no variations between monitoring and recovery groups in the thymus, spleen, and kidney, which are consistent with the study on an immunomodulatory efficacy of fenugreek and *Salacia chinensis* extracts in mice [18,22]. The hematological parameters of an animal are revealed and determined as one of the first immune responses, which are well balanced by the use of immunomodulating agents or natural compounds, as evident from the total leukocyte count and the differential count. The best described by the fact that the entrance of non-self-materials into the body is responded by blood cells, and the leukocyte count and differential counts are changed if the right protection is not imposed, as shown by CAME and PTME in our experiments [22]. Apart from these, the optimum dose is necessary for a potent immune response that makes a balance of hematological parameters, as explicit in our study by the more robust immune response asserted by the high neutrophil count [18].

After sensitization of mice with SRBCs, the macrophages released SRBC antigen, which was then processed. The B cells are stimulated when a T lymphocyte comes into contact; the B cell divides, expands, and differentiates, culminating in an antibody clone released by plasma cells [23]. As a result, the antibody links to the antigen, making it available for leukocytes to consume. Antibodies are glycoproteins that are members of the immunoglobulin protein family and consist of two big chains and two short medium chains [24]. Antibodies come in two varieties: a soluble form generated by the cell and a membrane-bound type linked to the outside of B cells. B cell receptor is a protein found mostly on the outside of B cells. It helps in the stimulation of these lymphocytes and their eventual metamorphosis into either antibody-producing plasma cells or B lymphocytes, which linger in the body and remain antigen-specific, allowing B cells to respond more quickly when antigens are presented [18]. To aid in the hunt for invading pathogens, the bloodstream and tissue fluids became densely packed with floating antibodies and particular releases. It appears that macrophages, T cells, and B lymphocytes, all involved in antibody generation, are particularly vulnerable. According to the current analysis, the extract improved the perfusate’s overall lymphocyte count compared to the control sample. The migration of leucocytes is essential for transporting immunological information between immune system compartments [25]. In this study, both experimental doses increased the neutrophil and lymphocyte counts in the latest analysis of differential leucocyte mobilization. It may be the product of a more robust immune system. In the early phases of acute inflammation, such as that produced by bacterial infection, chemical exposure, and certain malignancies, neutrophils are generally present in the circulation [26].

Inflammatory cells such as neutrophils are among the first to move to the inflammation site [27]. Chemotaxis is the process by which cells pass through the bloodstream and then into the interstitial in response to biochemical signals like interleukin-8 and C5a (complement component protein). The recruitment of neutrophils marks acute inflammation at the site of the damage within minutes of the trauma [28]. In this study, the number of monocytes, eosinophils, and basophils in the extract-treated group reduced compared to the control group. These cells are more vulnerable to immunomodulatory cytokines and pro-inflammatory chemicals, both of which are more prevalent in inflammatory environments. Eosinophils, basophils, and Th2 lymphocytes are also attracted to the inflammatory sites and are direct leukocyte receptors of IL 33 (Interleukin 33), suggesting that these innate immune cells are important in IL-33 biology. Basophils can play a role in anti-parasite immune reactions by being activated by immune serum and producing cytokines [29].

The mice’s paw swelling of CAME and PTME of the lowest doses was lesser than the paw thickness of a higher dose after 24 h of sensitization. It means that the extracts at a lower dosage suppressed the delayed hypersensitivity response. The anti-inflammatory characteristics of the extract are most likely responsible for its ability to suppress the delayed hypersensitivity reaction in this trial. Rather than antibodies, T cells and monocytes/macrophages are involved in this response. The DTH response [30,31] is an important defense mechanism against intracellular pathogens such as mycobacteria parasites and fungi, and it also plays a role in tumor immunity and transplant rejection. Instead of an immediate hypersensitive response within 12 min of an antigen challenge, “delayed” refers to a subsequent cellular reaction that occurs 48–72 h after antigen exposure. DTH necessitates activated T lymphocytes to identify a particular antigen, which proliferates and releases cytokines [31]. 

Plant extracts have been shown to activate macrophages. The main line of protection against pathogens is phagocytosis and macrophage destruction of invading microorganisms. Macrophages are phagocytic, microbicidal, and tumoricidal immune effector cells that play a critical function in the immune system. Macrophages play a significant part in activating and modulating immune responses by interacting with lymphocytes [18]. As usual, in this study, the lower dose of all extracts was found to have a higher PI. The current study’s results indicate that tested extracts have a significant immunostimulatory effect. It has been reported to have potent antioxidant, anti-inflammatory, and other medicinal properties. This result may be the effects of different phenolic and flavonoids compounds that may be the most plausible candidates for prompting an immunostimulatory impact. Several studies on medicinal orchids have yielded similar results [32,33]. Some of the abundant GC-MS (Gas Chromatography-Mass Spectrometry) analyzed compounds such as 9,12-octadecadienoic acid (Z,Z)-, eugenol and stigmasterol are already reported to show immunomodulatory function and our research is consistent with the previous results [34,35]. 

## Conclusion

According to the findings of this study, when the methanolic extracts of tested orchid species are given, the immune response is significantly improved. Furthermore, as observed, low doses of the orchid extract can trigger a cellular immune response better than the higher dose. The immune system’s ability to mount a reaction is impaired at the highest doses or greater. According to herbal drug research, medicine can have varying effects depending on the dosage of the drug administered. As a result, the methanolic extract of samples may have a toxicological or immunological influence on the body’s physiological or immunological processes. Despite this, when the drug was screened at lower concentrations, it had an immunomodulatory impact. Finally, the lower dose of CAME is better for strengthening the immune system than PTME. 

## List of abbreviations

CAME: *Cymbidium aloifolium* methanolic extract; PTME: *Papilionanthe* *teres* methanolic extract; SRBC: Sheep red blood cells; DHT: Delayed hypersensitivity test; PI: Phagocytic index; OECD: The organization for economic co-operation and development); DMSO: Dimethyl sulfoxide; PBS: Phosphate-buffered saline; WBC: White blood cell; RBC: Red blood cell; EDTA: Ethylenediaminetetraacetic acid; PMNs: Polymorphonuclear leukocytes; HBSS: Hank’s balanced salt solution; C5a: Complement component protein 5a; IL 8: Interleukin; IL 33: Interleukin 33; WP: White pulp; RP: Red pulp; GC-MS: Gas Chromatography-mass spectrometry; bw: Bodyweight; kg: Kilogram; gm: gram; min: minute; h: hour. 
